# Neural substrates involved in the cognitive information processing in teleost fish

**DOI:** 10.1007/s10071-021-01514-3

**Published:** 2021-04-27

**Authors:** R. Calvo, V. Schluessel

**Affiliations:** Institute of Zoology, Rheinische Friedrich-Wilhelms-Universität Bonn, Poppelsdorfer Schloss, Meckenheimer Allee 169, 53115 Bonn, Germany

**Keywords:** Cognition, Behaviour, Neuroanatomy, Learning, Neuroethology, IEG, Lesion studies

## Abstract

Over the last few decades, it has been shown that fish, comprising the largest group of vertebrates and in many respects one of the least well studied, possess many cognitive abilities comparable to those of birds and mammals. Despite a plethora of behavioural studies assessing cognition abilities and an abundance of neuroanatomical studies, only few studies have aimed to or in fact identified the neural substrates involved in the processing of cognitive information. In this review, an overview of the currently available studies addressing the joint research topics of cognitive behaviour and neuroscience in teleosts (and elasmobranchs wherever possible) is provided, primarily focusing on two fundamentally different but complementary approaches, i.e. ablation studies and Immediate Early Gene (IEG) analyses. More recently, the latter technique has become one of the most promising methods to visualize neuronal populations activated in specific brain areas, both during a variety of cognitive as well as non-cognition-related tasks. While IEG studies may be more elegant and potentially easier to conduct, only lesion studies can help researchers find out what information animals can learn or recall prior to and following ablation of a particular brain area.

## Introduction

Behavioural studies over the last few decades have shown that fish possess cognitive abilities greatly exceeding those originally suggested by Tinbergen, who stated that cognitve skills in fish are confined to fixed action patterns (Tinbergen 1951). Instead, there is ample evidence that fish possess cognitive abilities rivalling those of mammals and birds (for reviews see Brown et al. 2011; Schluessel 2015). ‘Cognition’ hereby refers to higher order mental functions (Brown et al. 2011; Marchetti 2018), that include four different processes: perception, attention, memory formation and learning (Brown et al. 2011; Shettleworth 2010; Schluessel 2015). Together, these four processes provide animals with the ability to make decisions (Shettleworth 2010; Ebbesson and Braithwaite 2012). Animal cognition is a rather modern field of research, aiming to comprehend animals’ mental abilities, as well as examining their underlying neural processes and mechanisms. There are several reasons that make fish a particularly interesting group to study this topic in. The group holds some of the most ancient forms of vertebrates, giving them a key position in the vertebrate phylogenetic tree. Compared to other vertebrates, there is also an unparalleled diversity featuring many exciting radiations which allow researchers to study influences of phylogeny versus ecology.

While cognition studies on fish are still less abundant than on mammals or birds—specifically in regards to the number of species studied—there really is a plethora of behavioural cognition studies available (see for example Kotrschal et al. 1998; Bshary et al. 2002; Brown et al. 2011). Furthermore, many studies over the last century have investigated fish, and in particular teleost neuroanatomy, assessing brain structures and their functions as well as neural connections and pathways both on a gross and molecular level (e.g. Nieuwenhuys 1963; Northcutt 1978, 2011; Northcutt and Braford 1980; Northcutt and Davis 1983; Nieuwenhuys and Pouwels 1983; Nieuwenhuys and Meek 1990; Wullimann 1997; Hofmann 2001; Rodríguez et al. 2005; Salas et al. 2006; Ito et al. 2007; Ito and Yamamoto 2009; Hurtado-Parrado 2010; Rupp et al. 1996; Vernier 2017; Yamamoto and Bloch 2017). Information about fish neuroanatomy is crucial to a deeper understanding of fish cognition as a whole, as cognitive input is processed in various regions throughout the brain. It has been suggested that fish neural architecture involved in cognitive information processing represents both analogous and potentially homologous structures to those found in mammals (Broglio et al. 2003, 2011), thereby supporting the behavioural findings that fish possess higher cognitive capabilities comparable to those of mammals including those of non-human primates (Brown et al. 2011). Unfortunately, only few studies have combined the two fields, i.e. behaviour and neuroanatomy, and identified the neural substrates involved in the specific processing of cognitive information in fish (e.g. Rodriguez et al. 2006; Kotrschal et al. 2013a, b; for reviews see Wullimann and Mueller 2004; Broglio et al. 2011; Ebbesson and Braithwaite 2012; Demski 2013; Maruska and Fernald 2018). In the following review, two approaches (lesions and IEG studies) that can be used to elucidate specific structure–function relationships involved in processing cognitive information will be presented. A third method, i.e. volumetric studies, will only be mentioned briefly, despite being frequently used to determine functionality from structure. Volumetric studies look for correlations between the presence or the extend of a particular ability and the size of a particular brain structure, which from our perspective, provides a worthy first step in identifying potential areas of interest but generally leaves more room for error than alternative methods. In-vivo imaging studies and optogenetic studies are also mentioned briefly at the end of the review, both offering exciting new possibilities. Lesions studies determine specific impairments in cognitive abilities following the removal of a particular brain region or target nuclei, while IEG studies look for differences in gene expression patterns in response to varying treatments, such as learning, stress or recalling of cognitive information compared to untreated controls. Both methods have advantages and disadvantages that will be discussed. To familiarize the reader with the teleost brain, a short overview of potentially relevant brain structures is provided first.

## The teleost brain

In the following, the cichlid brain will be introduced briefly for reference purposes. Obviously, fish brains vary in size and structure, and this section is only meant to provide a short overview of the major brain regions and nuclei that will be mentioned in later sections, i.e. in the lesion and IEG study descriptions, several of which have been conducted on cichlids. For this reason, any areas/nuclei mentioned throughout the paper (in any species) will be crosslinked to the figures provided in this section on the structure of the cichlid brain.

Generally, the fish brain follows the common vertebrate Bauplan. The neural tube gives rise to three primary morphological vesicles: the forebrain (prosencephalon), the midbrain (mesencephalon), and the hindbrain (rhombencephalon), which is continuous with the spinal cord. The three vesicle stage develops into the five-vesicle stage. As described in the “columnar model”, the forebrain— which in fish lacks the mammalian neocortex—is subdivided into the diencephalon caudally and the telencephalon rostrally. The diencephalon is then further divided into the thalamus dorsally and the hypothalamus ventrally, while the telencephalon is further subdivided into the pallium dorsally and the subpallium ventrally. The midbrain connects the forebrain to the hindbrain (rhombencephalon). Finally, the hindbrain is divided into the myelencephalon (containing the medulla oblongata) caudally and the metencephalon (containing the cerebellum and pons) rostrally (Herrick 1910; Wullimann 1997; Simões et al. 2012; Yamamoto and Bloch 2017).

A second model, the “prosomeric model”, was proposed by Puelles and Rubenstein in the early 1990s and attributes morphological meaning to gene expression patterns (Puelles and Rubestain 2003). Here, the forebrain is subdivided into the posterior diencephalon and the anterior secondary prosencephalon. The diencephalon is then further subdivided into the pretectum, thalamus, and prethalamus, while the secondary prosencephalon at the anterior end of the forebrain contains the telencephalon dorsally and the hypothalamus ventrally.

**Fig. 1:**
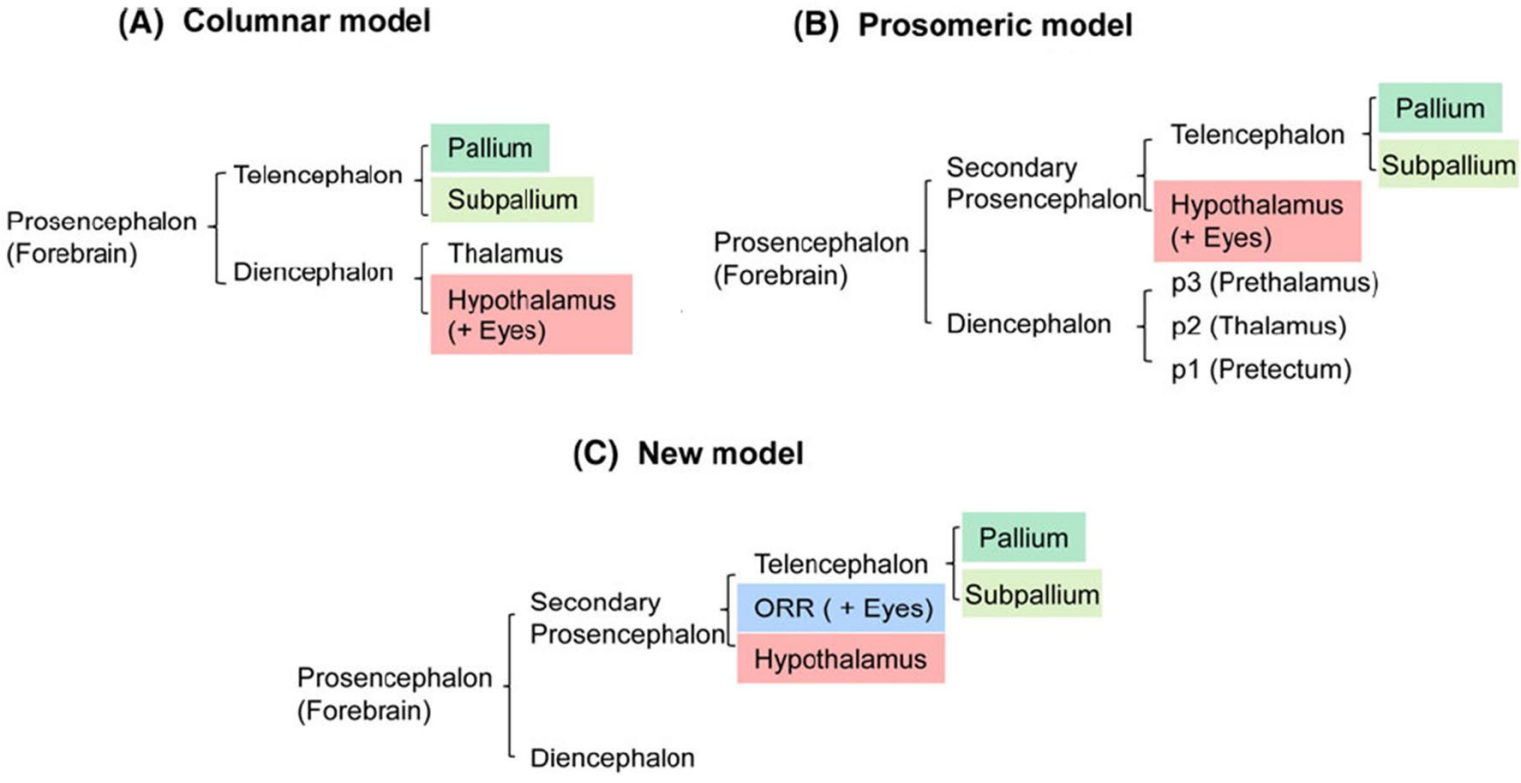
Models featuring the different subdivisions of the forebrain. **a** The columnar model in which the hypothalamus is considered to be the ventral half of the diencephalon. **b** The prosomeric model originally proposed by Puelles and Rubenstein in which the hypothalamus is proposed to be the ventral half of the most anterior part of the forebrain, and the telencephalon and hypothalamus consist of the secondary prosencephalon. **c** A new model proposed by Affaticati et al. in which the secondary prosencephalon is divided into three parts, the telencephalon, hypothalamus, and optic recess region (ORR) ( modified from Yamamoto et al. 2017)

**Fig. 2:**
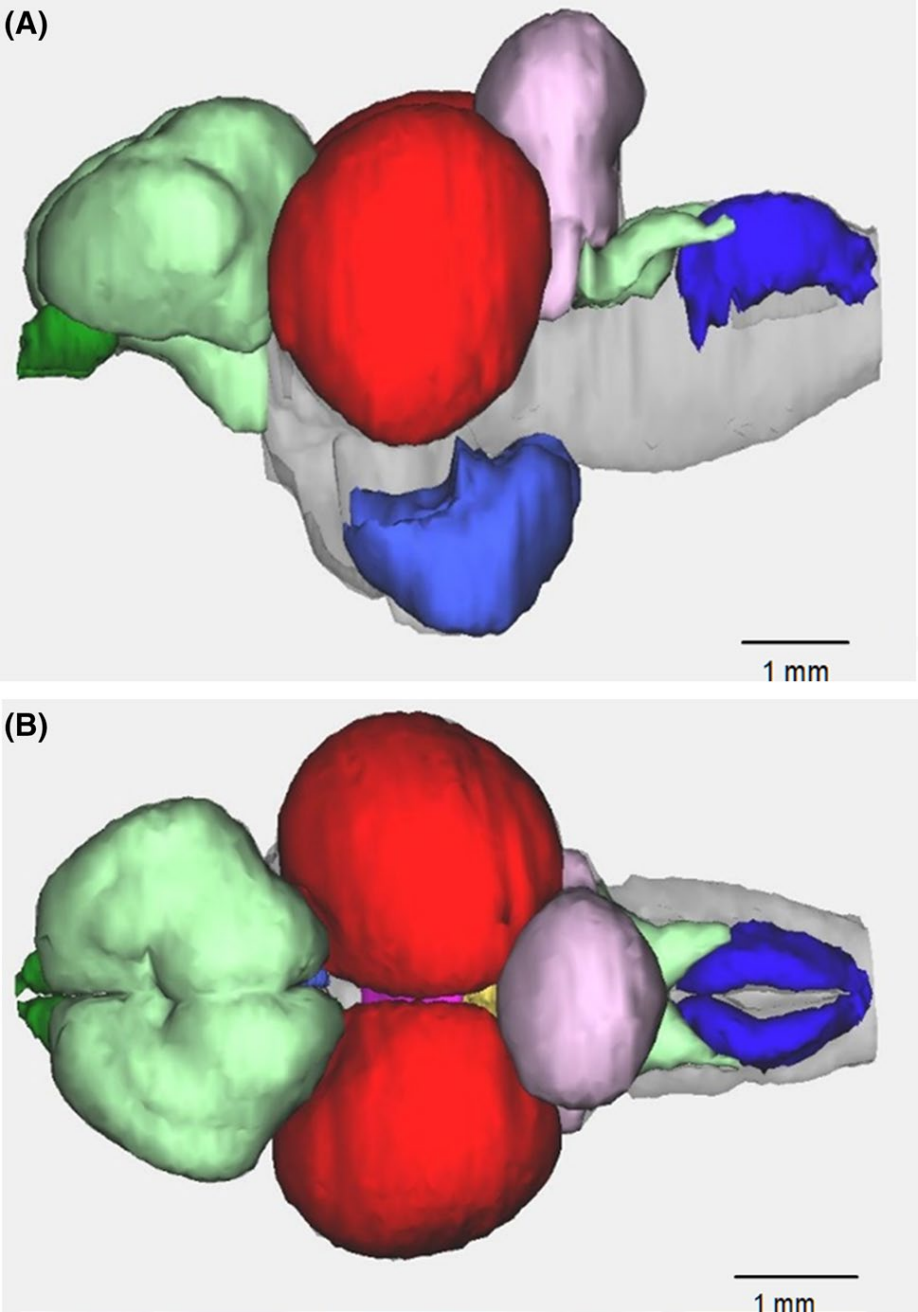
Brain of a cichlid fish, *Thorichtys meeki*. **a** Lateral view; **b** dorsal view. Six major areas can be identified: olfactory bulbs (dark green), telencephalon (light green), optic tectum (red), cerebellum (pink) + crista cerebellari (light green), inferior lobe of hypothalamus (purple), vagal lobe (blue) (courtesy of Michael Hofmann) (colour figure online)

The third and newest model, proposed by Affaticati et al. (2015), divides the secondary prosencephalon into three parts: the telencephalon, hypothalamus, and optic recess region (Affaticati et al. 2015). Figure 1 presents the three available models featuring the different subdivisions of the forebrain (Yamamoto et al. 2017 modified), Fig. 2 shows the major brain regions of a teleost fish from a dorsal and lateral perspective.

**Fig. 3:**
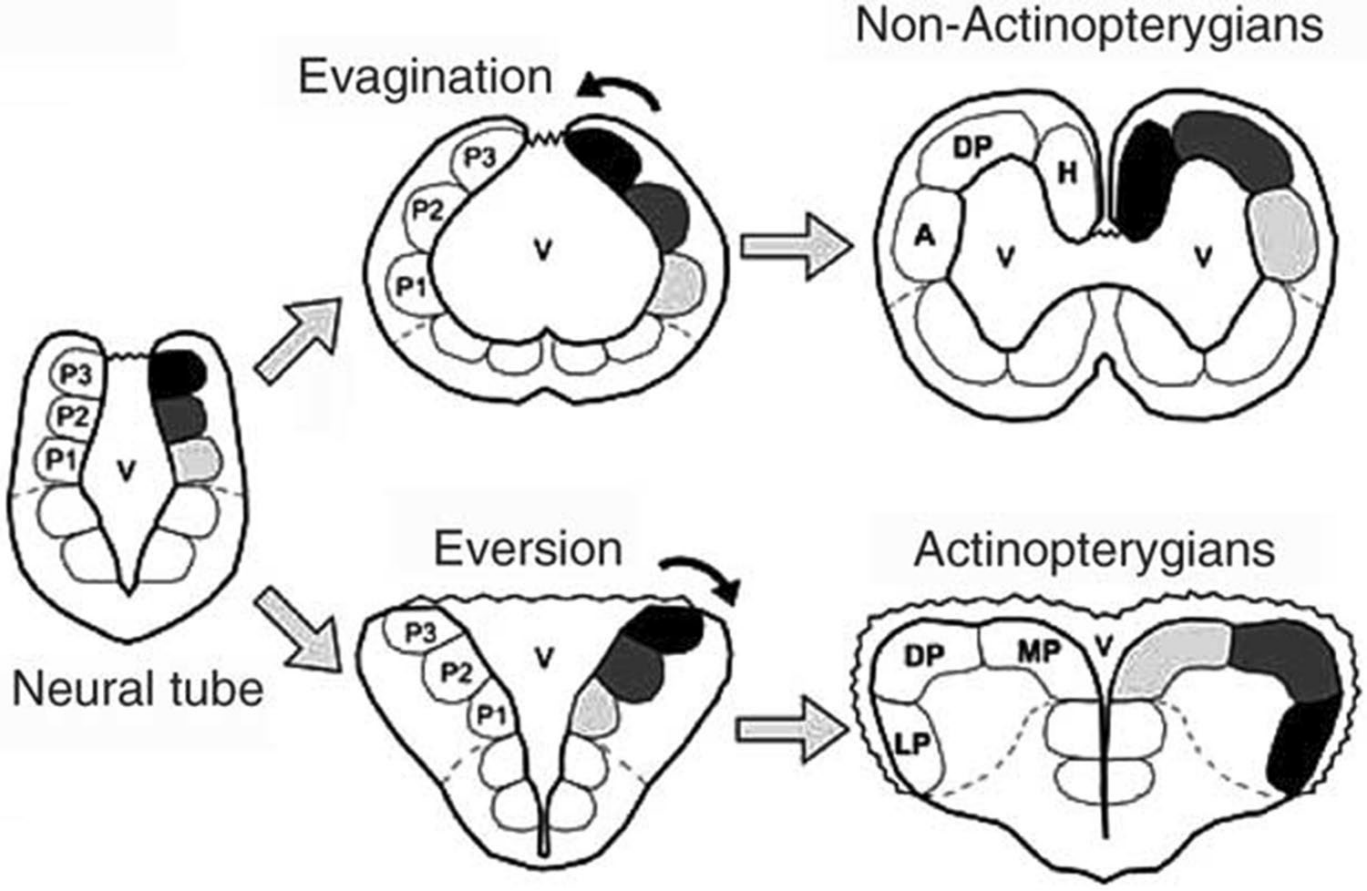
Schematic representation of the process of evagination in Non-Actinopterygians and eversion in Actinopterygians (modified from Broglio et al. 2005)

The telencephalon of Actinopterygians (which represent the largest group within the fishes) undergoes a different embryological development than all other craniates (Fig. 3), a so-called ‘eversion’ process (Gage 1893) that produces two telencephalic hemispheres separated by a single ventricle (Broglio et al. 2005) and a proliferative zone that lies at the dorsal part of the telencephalon (Mueller and Wullimann 2009). All other craniates undergo an ‘evagination’ process that produces two telencephalic hemispheres, each one with its own ventricular cavity, and a proliferative zone that is oriented towards the ventricles (Muller and Wullimann 2009).

Due to the different embryological development of Actinopterygians, potentially homologous or functionally equivalent structures are found in different locations than in other fish (such as in the chondrichthyans) or other vertebrate groups (Wullimann 1997). The forebrain of ray-finned fishes contains a number of areas common to all species (Northcutt 2002) while other areas are only seen in some highly derived groups, such as cichlids. This can include the presence or absence of nuclei or different structuring of particulate regions.

**Fig. 4:**
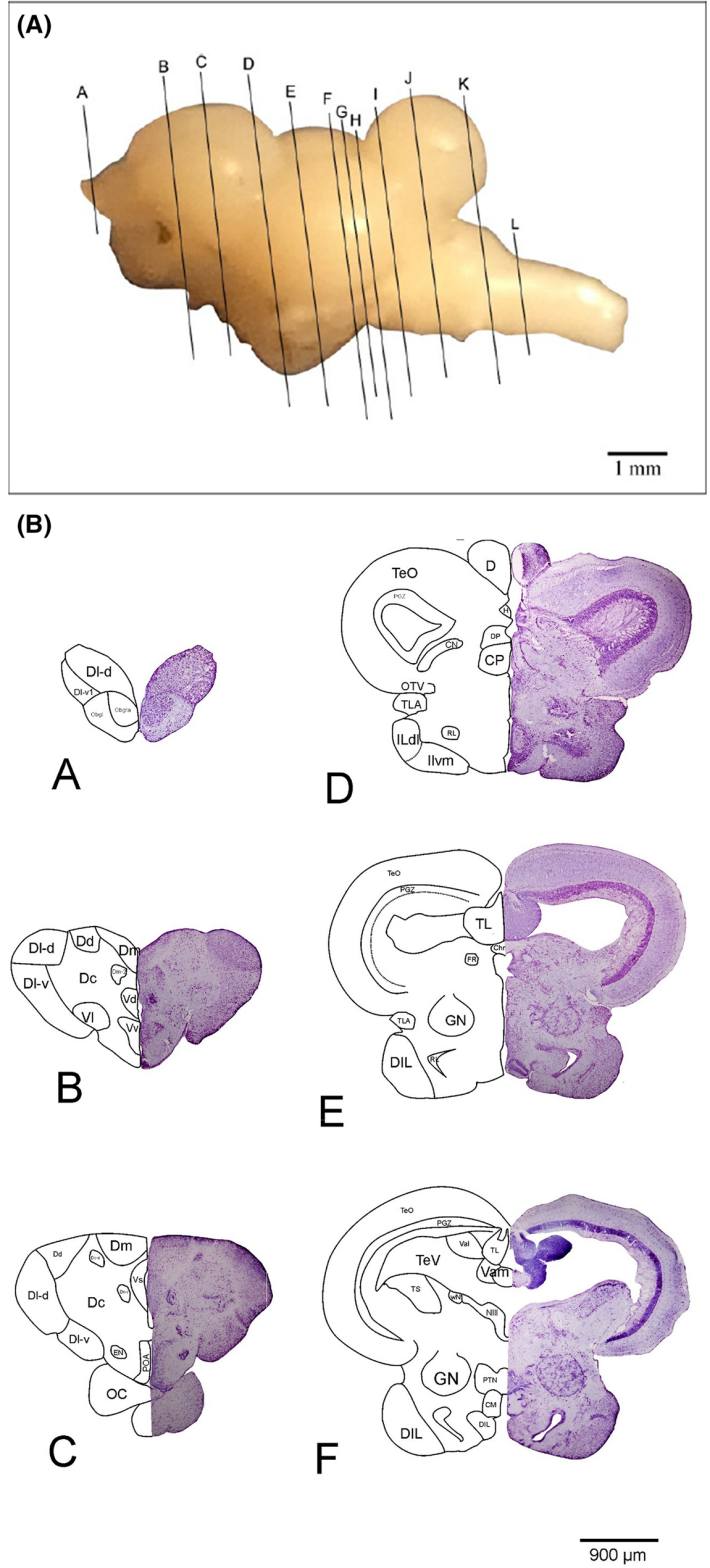

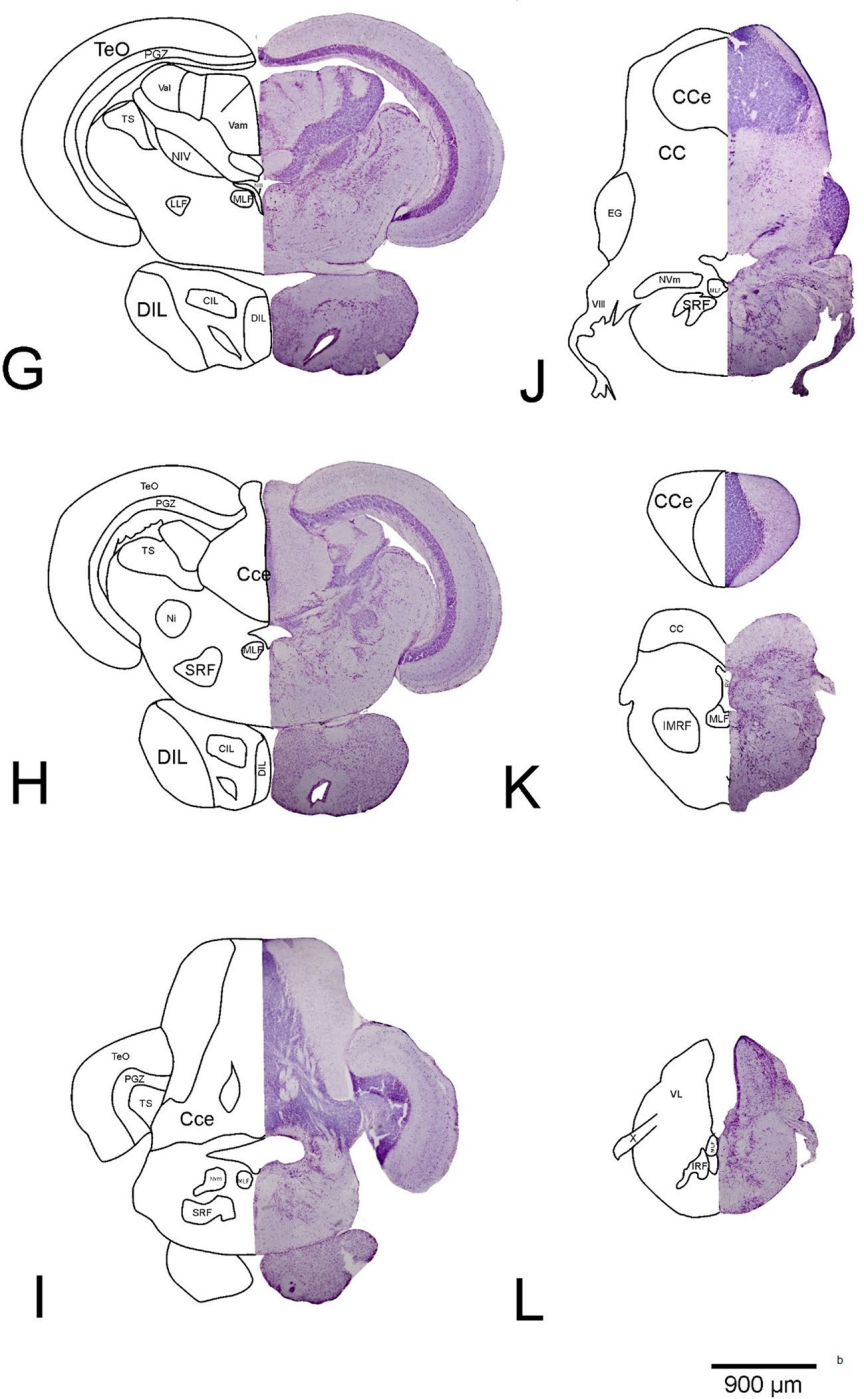
**a** Shown is the lateral view of the *Pseudotropheus zebra* brain (rostral to caudal); the oblique lines (A–L) give the locations of the transverse sections through the brain. **b** Cryostat sections of the P. zebra brain (A–L). The right side shows the microphotos of the original sections after Nissl staining, the left side shows a schematic drawing of identifiable areas and nuclei. *CC* crista cerebellaris, *CCe* corpus cerebelli, *Chr* commissura horizontalis, *CIL* central nucleus of inferior lobe, *CM* mamillary body, *CN* nucleus corticalis, *CP* central posterior thalamic nucleus, *D* dorsal telencephalic region, *Dc* central area of D, *Dc-d* dorsal subdivision of Dc, *Dc-r* rostral area of Dc, *Dc-v* ventral division of Dc, *Dd* dorsal area of D, *DIL* diffuse nucleus of the inferior lobe, *Dl* lateral area of D, *Dl-d* dorsal subdivision of Dl, *Dl-g* granular area from Dl, *Dl-v* ventral subdivision of Dl, *Dl-v1,2* parts of Dl-v, *Dm* medial area of D, *Dm-1,2,3* subdivisions of Dm, *DP* dorsal posterior thalamic nucleus, *Dp* posterior area of D, *dpca* decussation of anterior cerebellar peduncle, *EG* eminentia granularis, *EN* entopeduncular nucleus, *FR* fasciculus retroflexus, *GN* nucleus glomerulosus, *H* habenula, *ILdl* dorsolateral part of the inferior lobe, *ILvm* ventromedial part of the inferior lobe, *ILdv* ventromedial part of the inferior lob, *IMRF* intermediate reticular formation, *IRF* inferior reticular formation, *LFB* lateral forebrain bundle, *LLF* lateral-longitudinal fasciculu, *MLF* medial-longitudinal fasciculus, *Mo* medulla oblongata, *MS* spinal cord, *Ni* nucleus isthmi, *NIII* nucleus nervi oculomotori, *NIV* trochlear nucleus, *NVm* motor nucleus of trigeminal nerve, *Ni* nucleus isthmi, *NPT* posterior tuberal nucleus, *OB* olfactory bulb, *Obgl* glomerular area of the olfactory bulb, *Obgra* granular area of the olfactory bulb, *OC* optic chiasm, *OTV* ventrolateral optical tract, *PGZ* periventricular gray zones of the TeO, *POA* pre-optic area, *PTN* nucleus posterior tuberis, *RL* lateral recess, *RV* rhombencephalic ventricle, *SRF* superior reticular formation, *TTB* tectobulbaric tract, *Tel* telencephalon, *TeO* optic tectum, *TeV* tectal ventricle, *TL* torus longitudinalis, *TLA* lateral torus, *TOd* dorsal optic tract, *TOv* ventral optic tract, *TS* semicircular torus, *V* ventral telencephalic area, *VIII* vestibulocochlear nerve, *Vam* medial area of the valvula cerebelli, *Val* lateral area of the valvula cerebelli, *Vc* central area of V, *Vd* dorsal area of V, *VL* vagal lobe, *Vl* lateral area of V, *Vs* supracommissural nucleus of V, *Vv* ventral area of V, *WN* Edinger-Westphal Nucleus, *X* vagus nerve (slides taken from Jauch 2015)

The neural correlates for most cognitive functions in fish are still largely unknown, with the well-known exception of the lateral and medial divisions of the dorsal telencephalon (see Fig. 4b, section D). These two areas have been investigated in several studies and are by many considered to be potential homologues of the mammalian hippocampus and amygdala, respectively (for reviews see Rodriguez et al. 2006; Broglio et al. 2011; Ebbesson and Braithwaite 2012; but see also Saito and Watanabe 2004; 2006). However, these areas may also be involved in additional, so far unstudied, cognitive processes. Moreover, the functions of other areas within the telencephalon, such as the dorsal or central divisions of the dorsal telencephalic area (see Fig. 4b, sections B, C), are still unknown, as are the functions of most of its ventral regions.

Even less information is available on the diencephalon or the cognitive involvement of brain regions outside of the forebrain. While the connectivity between and within some brain areas may be known (e.g. Ahrens and Wullimann 2002; Folgueira et al. 2004a, b), there is not a single nucleus or area in the diencephalon whose function or contribution to possessing specific cognitive abilities has been studied and identified in detail. This includes the habenula (see Fig. 4b, section D), the thalamus (see Fig. 4b, section D) and the hypothalamus, which is highly derived in many teleost groups (Ahrens and Wullimann 2002). There is a prominent visual pathway extending from the tectum over the nucleus corticalis and the nucleus glomerulosus (see Fig. 4b, sections E, F) to the inferior lobes of the hypothalamus (see Fig. 4b, sections F–H) (Wullimann and Meyer 1990; Butler et al. 1991; Ahrens and Wullimann 2002). There are also some other highly derived areas, such as the mammillary body (see Fig. 4b, section F) and the nucleus of the posterior tuberculum. The latter has extensive projections to the medial part of the dorsal telencephalon (Murakami et al. 1983). In some fish groups, other areas are also markedly elaborated, e.g. the lateral torus (see Fig. 4b, sections D, E), which is related to gustatory functions (Ahrens and Wullimann 2002), and the anterior tuberal nucleus, which is particularly enlarged in catfish, and possibly related to acoustic communication. A range of forebrain areas has been electrically stimulated and behavioural responses have been described for several fish species (e.g. Demski 1973, 1977, 1983; Demski and Knigge 1971; Demski and Picker 1973). Stimulations in a number of hypothalamic areas for example changed specific types of social behaviours, suggesting that some social functions are located or mediated by the hypothalamus. Particularly the inferior lobes (see Fig. 4b, sections G, H) can be quite large and receive indirect visual input through the nucleus glomerulosus (see Fig. 4b, sections E, F). In some species, the inferior lobes can be even larger than the entire telencephalon (Hofmann, pers. comm.). In summary, only a few detailed studies, focusing on a few potential roles of specific forebrain areas, exist, while it seems highly likely that at least some of these regions are also involved in the processing of (other) cognitive tasks.

### Volumetric and lesion studies

There are many correlative studies linking brain size to cognitive ability, environment and/or ecology (e.g. Pike et al. 2018). Studies across mammals and birds have shown that overall brain size and cognitive abilities usually correlate positively (e.g. Reader and Laland 2002; Sol et al. 2005; Deaner et al. 2007). Also, closely related species occupying different niches may feature distinct differences in the size of major brain areas, related to differences in cognitive abilities (Tebbich and Bshary 2004). For example, higher cognitive abilities are required to find food in complex habitats compared to more simple or unstructured habitats. As a result, associated brain regions, e.g. the hippocampus (or its homologue) are enlarged in species that live and forage in complex environments (e.g. Sherry et al. 1989, 1992; Lucas et al. 2004). Similarly, brain size in fish may correlate with habitat, lifestyle or cognitive capabilities (e.g. Pollen et al. 2007; Salvanes et al. 2013; Northcutt 1977; Yopak et al. 2007; Yopak 2012; Pike et al. 2018). In cichlids, studies have linked brain complexity, size and volume with ecology, lifestyle or behaviour (e.g. Anken and Bourrat 1998; Pollen et al. 2007; Pollen and Hofmann 2008; Burmeister et al. 2009; Shumway 2010; Gutiérrez-Ibáñez et al. 2011). Environmental factors may permanently enhance learning abilities of fish (Kotrschal and Taborsky 2010). In guppies, a larger overall brain size was positively linked to cognitive differences (Kotrschal et al. 2013a,b,^2014^) and large-brained guppy females outperformed small-brained females in a reversal task but not in a colour discrimination test (Buechel et al. 2018). While these studies provide an important first step towards identifying relevant brain regions involved in cognitive information processing, correlative evidence is prone to errors and usually limited to assessing major brain areas as opposed to determining the detailed functioning of selected nuclei or smaller areas. Lesion studies, despite their own shortcomings (Lomber 1999), provide a more comprehensive method of determining structure–function relationships.

Pioneering lesion studies have focused on the involvement of the telencephalon but no other structures in cognitive processing (reviewed in Hofmann 2001; see also Savage 1980; Overmier and Curnow 1969; Overmier and Savage 1974; Laming and McKinney 1990) and detected impairments following ablation in some learning and memory functions (e.g. avoidance and spatial learning; e.g. Flood et al. 1976; Davis and Kassel 1983; Overmier and Hollis 1983; Rodriguez et al. 2006). Removal of the telencephalon (see Fig. 4b, section E) in teleosts does not seem to be as deleterious as in mammals (e.g. Kaas 1987; Hofmann 2001) and it is likely that cognitive functions are not all situated within the telencephalon. However, the only other brain structure that has been looked at in some detail in this context is the cerebellum (see Fig. 4b, sections J, K). It has been implicated in various conditioning tasks (Karamian 1963; Aronson and Herberman 1960; Álvarez et al. 2002; Gómez 2003) as well as avoidance or emotional learning (e.g. Kaplan and Aronson 1969; Álvarez et al. 2003; Gómez 2003, Yoshida et al. 2004; Rodríguez et al. 2005) and spatial cognition (Durán et al. 2004; Rodríguez et al. 2005).

A cognitive ability, relevant to most species and quite thoroughly investigated in many vertebrate and invertebrate species, is spatial cognition, which includes spatial learning and memory. Behaviours, such as orientation, navigation, migration or homing, depend on spatial cognition (Dodson 1988; Rajan et al. 2011). Spatial learning is directly connected to spatial memory as it allows an individual to record and recall information about its environment and its orientation, for example feeding or nesting locations. For this reason, spatial memory and spatial learning are essential both for animals that do not change habitat, as well as for animals migrating (Wood et al. 2011). In its most complex form, spatial cognition entails the ability of an organism to acquire a mental representation of the environment, i.e. to construct a cognitive spatial map (Glikmann-Johnston et al. 2015). In a series of studies on the goldfish (*Carassius auratus*), behavioral and detailed neuroanatomical approaches were used complementary to elucidate spatial abilities and neuroanatomical correlates (reviewed in Broglio et al. 2011). Results indicated that not the telencephalon as a whole, but specifically the lateral zone of the dorsal telencephalon (see Fig. 4b, sections A–C), considered to be a hippocampus homologue, plays a crucial role in complex place learning (allocentric orientation) and spatial memory in goldfish (e.g. Salas et al. 1996a, b; López et al. 2000; Durán et al. 2008, 2010; Costa et al. 2011). It was also established that egocentric spatial strategies are unlikely to be processed—at least exclusively—in the telencephalon (e.g. Salas et al. 1996a, b; López et al. 2000; Rodríguez et al. 2002). Results were contradicted by findings from Saito and Watanabe (2004,2006), who claimed impairments in the dorsomedial telencephalon (see Fig. 4b, sections B, C), instead of the dorsolateral part, to be responsible for the disruption of spatial abilities (for a critical discussion see Rodriguez et al. 2006). More recently, similar results to those obtained on the goldfish were observed in studies on the spatial abilities of sharks and stingrays (Schluessel and Bleckmann 2005, 2012; Fuss et al. 2014a, b). In sharks, the dorsomedial pallium, like the lateral pallium in teleosts, seems to play a crucial role in processing more complex place learning information (Fuss et al. 2014a) while not being implicated in the processing of egocentric information, i.e. turn procedures (Fuss et al. 2014b). Accordingly, as in the goldfish, different neural substrates seem to be responsible for different spatial functions and mechanisms in sharks. As suggested by Rodriguez et al (2006), results indicate that the dorsomedial portion of the pallium in sharks may be comparable to the hippocampus of land vertebrates and the lateral pallium of teleosts (Fuss et al. 2014a, b). In two blenniid species, sex-specific differences in regards to spatial demands were found to exist both behaviourally and neuronally (Costa et al. 2011). White and Brown (2015) tried to correlate volume measurements of various brain regions with spatial ability in two species of guppies with different ecological needs. Differences were found in the size of the telencephalon, the optic tectum and the hypothalamus as well as different spatial abilities between the two species.

 Very few studies are available assessing the involvement of neural substrates in cognitive behaviours other than spatial orientation. In goldfish, avoidance learning paradigms were investigated and the medial zone of the dorsal telencephalon in teleosts found to perform similar functions to the pallial amygdala of land vertebrates (reviewed in Rodriguez et al. 2006; Portavella and Vargas 2005; Portavella et al. 2002; Broglio et al. 2011). Similar results were observed in juvenile bamboo sharks (Schwarze et al. 2013).

Table 1 gives an overview of the most relevant lesion studies assessing neural structures in fish in a cognitive context.

**Table 1:** Overview of studies combining behavioral and neuroanatomical studies in fish

Brain region	Behavior	Author
Lateral zone of pallium	Spatial orientation	Salas et al. (1996a, b)
Dorsolateral telencephalon	Spatial orientation	Vargas et al. (2000)
Lateral zone of pallium	Spatial orientation	López et al. (2000), Durán (2004), Broglio et al. (2005)
Dorsomedial telencephalon	Spatial orientation	Saito and Watanabe (2004,2006)
Lateral pallium	Encoding spatial information	Vargas et al. 2006
Entire telencephalon	Spatial orientation	Durán et al. (2008)
Dorsolateral ventral telencephalic nuclei	Spatial orientation	Costa et al. (2011)
Pallium^a^	Spatial orientation	Fuss et al. (2014c)
Pallium^a^	Spatial orientation	Fuss et al. (2014d)
Telencephalon,hypothalamus, optic tectum	Spatial orientation	White and Brown (2015)
Cerebellum	Spatial orientation	Durán et al. (2004), Rodríguez et al. (2005)
Medial zone of pallium	Emotional memory/avoidance learning	Portavella and Vargas (2005), Portavella et al. (2002, 2004), Durán (2004)
Telencephalon	Avoidance learning	Schwarze et al. (2013), Overmier and Flood (1969), Overmier and Hollis (1990)
Telencephalon	Conditioning tasks and habituation	Savage (1980), Overmier and Curnow (1969), Overmier and Savage (1974), Laming and McKinney (1990)
Telencephalon, optic tectum, cerebellum, dorsal medulla, hypothalamus and olfactory bulb	Reversal learning/spatial learning	Fong et al. (2019)
Optic tectum, olfactory bulbs	Discrimination tasks	Pike et al. (2018)
Cerebellum	Conditioning tasks	Karamian (1963), Aronson and Herberman (1960), Álvarez et al. (2002), Gómez (2003)
Cerebellum	Emotional learning	Kaplan and Aronson (1967), Aovarez et al. (2003), Gómez (2003), Yoshida et al. (2004), Rodríguez et al. (2005)
‘Nuclei of the conserved social behavior network’	Social hierarchy*	Maruska et al. (2013)

Note that most studies have tried to look for spatial orientation correlates

*Not cognition studies per se, but studies of behaviors that include some cognitive aspects

^a^The medial pallium in elasmobranchs corresponds to the lateral zone of the pallium in teleosts due to different folding events during embryogenesis. We attempted to remove the medial pallium; however, as it was difficult to target almost the entire pallium was removed in most individuals

Lesion studies, where parts of the brain are ablated, can help researchers to identify the neural substrates involved in cognitive information processing by testing what animals can do prior to and following surgery. The most obvious shortcoming of this technique is that lesions are hard, if not impossible to place without damaging non-target tissue ‘on route’ to the target destination. While electrodes of micromanipulators are extremely fine, the extent of damage created is still difficult to estimate. Additional problems are to actually ‘find’ the correct target area, finding the same area repetitively in different individuals (also of varying sizes), destroying a significant portion of a relevant area and rendering it unfunctional in the process, and making sure that the lesioned area is in fact responsible for the processing of a particular information as opposed to simply being part of a relevant pathway in the information transfer. Last but not least, one always has to consider further surgery effects, such as causing motivational, sensory or motor impairments that keep animals from performing at the same level as prior to surgery. For example, damaging the target area could cause a hyperactivation or hyperinactivation of other brain regions that, under normal conditions, are activated or inhibited by the damaged region (Fuster 1989; Damasceno 2010). Some of these shortcomings can be overcome using sham-operated and control animals, as well as lesioning a larger number of individuals (also to overcome intra-specific variation). Some, however, cannot be controlled for. Due to this circumstance, it seems ideal to combine lesion studies with an additional method, that has widely been used in recent years and from our perspective can make up for some of the problems encountered, immediate early gene analyses.

### Immediate early gene studies

The study of the expression of immediate early genes (IEGs) is a more recent but very promising method to investigate and visualize neuronal activity in the brain when investigating substrates underlying synaptic plasticity processes, such as long-term potentiation (LTP), long-term depression (LTD) and cognitive functions (Minatohara et al. 2016). IEGs are cellular genes that are responsive to extracellular stimuli, more precisely, they are first response genes whose expression is regulated immediately after stimulation. Transcriptional activation of RNA occurs in the nucleus within five minutes of stimulation and continues for 15–20 min, after which the transcripts are transferred to the cytoplasm (Greenberg and Ziff 1984; Greenberg et al. 1985; Guzowski et al. 1999). Induction occurs within minutes and is short-lived; typically, IEG mRNA levels reach their maximum 30–60 min after stimulation and decline after 2–5 hrs to baseline. The protein concentration reaches its maximum about 60–90 min after stimulation and disappears within four hours of treatment (Curran and Morgan 1995).

Different studies conducted on PC 12 pheochromocytoma cells highlighted the involvement of IEGs in neuronal function (Sheng and Greenberg 1990; Morgan and Curran 1991; Curran and Morgan 1995). In conjunction with more recent research, these studies demonstrate that IEGs are expressed throughout the nervous system and that various types of stimulation (such as pharmacological agents, behavioral tests, seizures, etc.) can increase their expression (Curran and Morgan 1995). In fact, IEG expression is a crucial part of a neuron’s response to behaviourally relevant stimuli and codes for several classes of proteins displaying different functions, such as signalling molecules, postsynaptic proteins, metabolic enzymes, cytoskeletal proteins, growth factors or transcription factors (Lanahan and Worley 1998). There is also a correlation between a local increase in IEG expression and neuronal activity, i.e. IEG expression can serve as a marker for neuronal activity. This indicates which types of neurons were activated and, above all, in which area of the brain the activation took place (Long and Salbaum 1998). It is estimated that there are about 30–40 different IEGs that can be expressed in neurons. Of these, 10–15 could serve as regulatory genes, i.e. function as transcription factors (Lanahan and Worley 1998) capable of regulating the expression of target genes (named late-response genes) and influencing neuronal physiology (Curran and Morgan 1987; Curran and Franza 1988; Herdegen and Leah 1998; O’Donovan et al. 1999; Tischmeyer and Grimm 1999; Pinaud 2004; Pinaud et al. 2005; Gallo et al 2018). Among those IEGs that function as transcription factors, the most investigated genes for mapping activity in the brain are *c-fos* and *egr-1*. Both are involved in cell differentiation and proliferation and, most importantly, they serve a crucial function in cognitive processes, particularly in learning and memory, but also in synaptic plasticity in general (Okuno 2011).

### C-FOS

*C-fos*, whose induction was the first one among IEGs to be shown as activity-dependent (Morgan and Curran 1988; Sagar et al. 1988; Gallo et al. 2018) belongs to the Fos family and is a protooncogene (Morgan and Curran 1989). It encodes the nuclear C-FOS protein, a 62-kDa product which undergoes post-translational modifications that mainly consist of serine and threonine phosphorylation (Curran et al. 1984; Barber and Verma 1987). Furthermore, *c-fos* can negatively regulate its own expression and this characteristic is required for a rapid decline in its expression (Morgan and Curran 1991). In neurons, the first detailed studies assessing the regulatory mechanisms of IEGs were performed on *c-fos* (Schilling et al. 1991; Sheng et al. 1990; Okuno 2011) and, under baseline conditions, there is little or no expression of this gene in most neurons (Morgan and Curran 1989; Hoffman et al. 1993). The expression of various late-response genes involved in different neuronal processes (for example growth control or plastic changes) is induced by the activation of *c-fos* gene (Sukhatme et al. 1988; Williams et al. 2000; Bozon et al. 2003; Maddox et al. 2011; Gallo et al. 2018).

### EGR-1

The gene *egr-1* is also known as *zif/268*, *krox-24*, *TIS8*, *NGFI-A* or *zenk*; it codes for a transcription factor (*Egr-1*) that is a member of a four-gene family of *Egr* and also plays an important role in neural plasticity during neuronal activation through sensory stimulation. *Egr-1* is a phosphorylated protein and it is synthesized in the nucleus, where it remains thereafter (Cao et al. 1990). Furthermore, this protein has the ability to autoregulate its own expression (D. Gius, X. Cao, and V. P. Sukhatme, unpublished, referenced in Cao et al. 1990). The *Egr-1* expression in the brain is specific to neurons and its activity is strongly (but not exclusively) regulated by synaptic activity (Worley et al 1991). *Egr-1* expression is continually induced by ongoing synaptic activity (Burmeister and Fernald 2005) as a consequence of the basal physiological synaptic activity (Worley et al. 1991). This is unlike other, similar inducible transcription factors (such as *C-fos*), whose expression declines after the initial stimulation (Herdegen et al. 1995; Kaczmarek and Chaudhuri 1997). It is still unclear which are the targets that *egr-1* regulates under physiological stimulation in vivo, but synapsins (Petersohn et al. 1995; Thiel et al. 1994; Burmeister and Fernald 2005) and neurofilaments (Mello 2004; Burmeister and Fernald 2005) are two likely candidates (Burmeister and Fernald 2005).

To investigate the evolutionary conservation of *egr-1*, cichlid *egr-1* (*Astatotilapia burtoni*) was cloned by Burmeister and Fernald (2005) and its protein sequence compared to available representatives of other vertebrate groups. They demonstrated that *A. burtoni egr-1* shares 81% sequence similarity with zebrafish (*Danio rerio*) and 66% with mouse (*Mus musculus*) *egr-1*. This has been the only characterization of *egr-1* in a vertebrate other than a mammal or bird (Burmeister and Fernald 2005). Another important discovery coming out of this study showed that the *egr-1* expression kinetics is similar to the one of mammals (Zangenehpour and Chaudhuri 2002) and birds (Mello and Clayton 1994) by reaching its highest expression levels 30 min after stimulation (Burmeister and Fernald 2005).

The following provides a summary of previous research and advances that have successfully used IEGs as markers of cognitive processing in fish (see Table 2).

**Table 2:** Overview of studies combining behavioral and IEGs studies in fish

Brain region	IEGs	Behavior	Author
Pre-optic area, medial zone of the dorsal telencephalon, ventral subdivision of the lateral zone of the dorsal telencephalon	c-fos egr-1	Social habituation	Weitekamp et al. (2017)
Telencephalon, hypothalamus, cerebellum	egr-1	Alloparental-care opportunity and submissive behavior	Kasper et al. (2018)
Nuclei of social decision-making network (SDMN): ventral nucleus of ventral telencephalon, rostral portion of the ventral nucleus of the telencephalon, supracomissural nucleus of the ventral telencephalon, dorsal part of the ventral telencephalon, anterior tuberal nucleus, anterior part of the periventricular preoptic nucleus, parvocellular division of the magnocellular preoptic nucleus, magnocellular division of the magnocellular preoptic nucleus, lateral nucleus of dorsal telencephalon, granular region, the posterior nucleus of the dorsal telencephalon	c-fos	Social behavior	Field and Maruska (2017)
Telencephalon, optic tectum, cerebellum	egr-1 c-fos	Social behavior	Sadangi (2012)
Nuclei of social behavior network (SBN): lateral part of the dorsal telencephalon, medial part of the dorsal telencephalon, ventral nucleus of the ventral telencephalon, supracommissural nucleus of the ventral telencephalon, preoptic area, ventral tuberal nucleus, anterior tuberal nucleus, corpus cerebellum	egr-1 c-fos	Social behavior—social hierarchy	Maruska et al. (2012)
Telencephalon and hypothalamus	egr-1 bdnf	Early social behavior	Nyman et al. (2017)
SDMN	egr-1 c-fos	Social behavior—aggressive behavior	Almeida et al. (2019)
Dorsolateral telencephalon, dorsomedial telencephalon, ventral telencephalon, ventral hypothalamus, central gray area, pituitary gland, nucleus preopticus, anterior tuberal nucleus, ventral sopracommissural telencephalon, cerebellum	egr-1	Mating behavior	Wong et al. (2012)
Telencephalon, optic tectum, hypothalamus, pituitary gland, cerebellum, medulla oblongata and the anterior part of the spinal cord	c-fos	Mating behavior	Okuyama et al. (2011)
Whole brain	egr-1	Mating behavior	Cummings et al. (2008)
Medial part of the dorsal telencephalon, the supracommissural nucleus of the ventral telencephalon, the ventral nucleus of the ventral telencephalon, the preoptic area	c-fos	Social buffering	Faustino et al. (2017)
Medial octavo lateralis nucleus, ventro lateral portion of the torus semicircularis, central portion of the torus semicircularis, central posterior thalamic nucleus, posterior part of the dorsal telencephalon SDMN regions: anterior tuberal nucleus, central part of the dorsal telencephalon, granular zone of the lateral zone of the dorsal telencephalon, magnocellular preoptic nucleus magnocellular division, magnocellular preoptic nuclus parvocellular division, periventricular nucleus of the posterior tuberculum, caudal subdivision of the dorsal part of the ventral telencephalon, supracomissural nucleus of the ventral telencephalon, ventral portion of the ventral telencephalon	c-fos	Social behavior—social interaction	Butler and Maruska (2016)
Dorsal telencephalon (lateral, medial, central)	egr-1	Social behavior—individual recognition	Harvey-Girard et al. (2010)
Anterior preoptic area	egr-1	Social behavior—social hierarchy	Burmeister et al. (2005)
Nuclei of SBN: lateral part of the dorsal telencephalon, medial part of the dorsal telencephalon, ventral nucleus of the ventral telencephalon, supracommissural nucleus of the ventral telencephalon, preoptic area, ventral tuberal nucleus, anterior tuberal nucleus, and corpus cerebellum	egr-1 c-fos	Social behavior—social hierarchy	Maruska et al. (2013)
Forebrain	c-fos	Social behavior—paternal care	O'Connell et al. (2012)
Telencephalon	egr-1	Visual discrimination	Fuss and Schluessel (2018)
Olfactory bulb	c-fos	Behaviors evoked by odorants	deCarvalho et al. (2013)
Medial zone of the dorsal telencephalic region, dorsal nucleus of the ventral telencephalic area dorsal thalamus (anterior nucleus, dorsal posterior thalamic nucleus, central posterior thalamic nucleus), hypothalamus, preglomerular nucleus, optic tectum, periventricular gray zone and in cerebellum	c-fos	Choice behavior	Lau et al (2011)
Social behavior network nuclei: medial amygdala, lateral septum, preoptic area, anterior and ventromedial hypothalamus, periaqueductal gray, dorsolateral telencephalon, cerebellum and raphe nucleus	egr-1 c-fos	Choice behavior	Desjardins et al. (2010)
Pre-optic area, lateral septum, anterior and ventromedial hypothalamus, periaqueductal gray, dorsomedial and dorsolateral telencephalon, cerebellum, raphe nucleus	egr-1 c-fos bdnf	Spatial cognition	Wood et al. (2011)
Telencephalon	egr-1	Spatial cognition	Rajan et al. (2011)
Dorsomedial telencephalon, dorsolateral telencephalon, preoptic area and cerebellum	egr-1 c-fos	Mirror image fighting	Desjardins and Fernald (2010)
SDM network nuclei: three subregions of the medial part of the dorsal telencephalon, one subregion of the supracommissural nucleus of the ventral pallium, the lateral subdivision of the lateral part of the dorsal telencephalon, parvocellular as well as magnocellular and gigantocellular cell groups of the preoptic area	c-fos	Social behavior—cooperative behavior	Weitekamp and Hofmann (2017)
Medial zone of the dorsal telencephalon, lateral zone of the dorsal telencephalon, ventral nucleus of the ventral telencephalon	egr-1 c-fos bdnf npas4	Emotion-like states	Cerqueira et al. (2017)
Central part of the dorsal telencephalon, ventral zone of the ventral region of the lateral part of the dorsal telencephalon, posterior part of the dorsal telencephalon, central part of the ventral telencephalon, ventral part of the ventral telencephalon, parvocellular and magnocellular subnuclei of the preoptic area	c-fos	Sensory integration of social signals	O’Connell et al. (2013)
Pre-optic area	egr-1	Aggressive and reproduction-related behaviours	Loveland and Fernald (2017)

Choice behaviour, i.e. the ability to make choices and perform actions and behaviours as a result of these choices, is critical for the survival of all individuals (Lau et al. 2011). To test this behaviour in fish, light avoidance, an innate choice behaviour, was examined in zebrafish (Lau et al. 2011). First, fish were introduced to a light/dark choice chamber. After giving them time to explore the environment, researchers found two distinct groups of animals: one composed of animals showing light-avoidance behaviour, and a second one that did not. These two different behaviours occurred regardless of whether the animals were initially placed on the dark or the bright side of the chamber. In situ hybridization analyses of the *c-fos* expression were performed and compared between the two groups. In the animals exhibiting light-avoidance behaviour, *c-fos* expression was detected in the medial zone of the dorsal telencephalic region (see Fig. 4b, sections B, C), potentially homologous to the mammalian amygdala. Another increase in *c-fos* expression was found in the dorsal nucleus of the ventral telencephalic area, possibly the teleost homologue of the mammalian striatum (Rink and Wullimann 2002). In the diencephalon, *c-fos* was detected in the hypothalamus and in different nuclei of the dorsal thalamus [anterior nucleus, dorsal posterior thalamic nucleus, central posterior thalamic nucleus (see Fig. 4b, section E)] and in the preglomerular nucleus [the last four nuclei are visually activated in the teleost brain (Wullimann 1997)]. Furthermore, *c-fos* expression was detected in the optic tectum (see Fig. 4b, sections G–L), in the periventricular grey zone (see Fig. 4b, sections E–K) and in the cerebellum (see Fig. 4b, sections J, K). In animals that exhibited low or no light-avoidance behaviour, the *c-fos* expression detected in the hypothalamus and in the visually related nuclei was similar to that of the 'avoidance group' but little *c-fos* was detected in the medial zone of the dorsal telencephalic region (see Fig. 4b, sections A–C) and in the dorsal nucleus of the ventral telencephalic area. The differential *c-fos* expression in the medial zone of the dorsal telencephalon and in the dorsal nucleus of the ventral telencephalon, and consequently the divergent activation of these regions, within the two groups of fish, led researchers to believe that these two regions may be involved in a circuitry that determines the performance of the light-avoidance behaviour. Furthermore, since the dorsal nucleus of the ventral telencephalon is “downstream” of the dorsal telencephalic region, it seems that the latter could play the role of a “choice centre” in this behaviour (Lau et al. 2011).

One of the most important decisions is choosing suitable mates or partners. The choice of a male partner by females, based on the information about male–male social interactions, was analysed by Desjardins et al. (2010) in *A. burtoni*. The study aimed to investigate specifically, which brain regions respond to visual information when choosing a mate. The expression of the two IEGs *c-fos* and *egr-1* was analysed in the proposed fish homologue of the brain nuclei of SBN (social behaviour network) in mammals (which includes the medial amygdala, the lateral septum, the preoptic area, the anterior hypothalamus and the ventromedial hypothalamus, the periaqueductal grey, the dorsolateral telencephalon, the cerebellum and the raphe nucleus) (Newman 1999). IEGs expression was compared in the brains of gravid females’ that, after having chosen a mate, witnessed a fight between the males of their choice. Females who had seen their respective preferred male win or lose a fight showed differences in IEG expression in all SBN brain nuclei. Additionally, differences in the level of *egr-1* and *c-fos* expression were found in other brain areas. More precisely, females who saw their preferred male win, had higher IEG expression in the ventromedial hypothalamus and in the preoptic area (see Fig. 4b, section C), known to be involved in reproductive behaviour. In the lateral septum, the expression of *c-fos* and *egr-1* was higher in females seeing their preferred male lose. The lateral septum is implicated in the modulation of anxiety-like behaviour, indicating that females seeing their respective male lose, could have experienced anxiety. In all other brain areas, there were no detectable differences in *egr-1* or *c-fos* between the two groups of females (Desjardins et al. 2010).

Wood et al. (2011) tested if *A. burtoni* could be trained in a spatial task and assessed if successful execution of the task was related to the expression of *c-fos*, *bdnf* and *egr-1* in the pre-optic area (see Fig. 4b, section C), lateral septum, anterior hypothalamus, ventromedial hypothalamus, periaqueductal grey, dorsomedial telencephalon, cerebellum (see Fig. 4b, sections J, K), raphe nucleus and the dorsolateral telencephalon (see Fig. 4b, sections A–C) (some of these nuclei were also included in the SBN, see above). Fish were divided in three groups (learners, non-learners and non-attempting) based on their performance in the task (finding a hole in a clear barrier that separated the tank in two compartments). In the dorsolateral telencephalon of learners, mRNA levels of both *bdnf* and *egr-1* were expressed at significantly higher levels than in non-attempting and non-learner fish, suggesting that the dorsolateral telencephalon may play a key role in spatial cognition. The lower activity of IEGs in the periaqueductal grey suggests lower stress levels in the learners than in non-learners and non-attempting fish. The preoptic area, playing a role in the reward and motivation pathway, also showed an increase in IEG expression, indicating increased motivation in learners over the training period. Overall, higher levels of IEG activity, a decreased stress response, and an increased motivation in learners suggest a heightened ability to learning a spatial task. In the brains of non-attempting fish, expression of *bdnf* and *egr-1* was increased in both the periaqueductal grey and in the dorsomedial telencephalon, indicating an activation within brain areas associated with anxiety and stress. Non-learners also exhibited lower levels of *bdnf* and *egr-1* within the dorsolateral telencephalon than learners. Furthermore, non-learners also exhibited lower levels of *egr-1* in the dorsolateral telencephalon, while featuring “intermediate” expression levels in the periaqueductal grey. In conclusion, learner fish showed high levels of activity within the area associated with learning and memory (dorsolateral telencephalon), no activity in areas associated with fear and stress (dorsomedial telencephalon and periaqueductal grey) and some activity in the preoptic area, indicative of high motivation (Wood et al. 2011).

In the same year, Rajan and colleagues examined whether spatial learning induces *egr-1* expression in the telencephalon (see Fig. 4b, sections A–C) of goldfish. Researchers divided a tank in four different compartments using three vertical transparent acrylic barriers and trained fish to pass through the barriers one by one. When fish had successfully accomplished the task, the third barrier was replaced by a modified one. Analysis showed that fish attempted more often to pass through the first barrier than the others, as the task was novel and the solution unknown, but already familiar when encountering the second and third barrier. *Egr-1* expression levels in the telencephalon were higher in a fish having mastered to cross the first barrier than in a resting control. However, the level of *egr-1* expression decreased again, when fish had learned to pass through barriers two and three. When the modified gate three was introduced, researchers observed an increased number of attempts correlating with an increased level of *egr-1* expression in the telencephalon. In conclusion, the study highlighted an increase in *egr-1* expression in the telencephalon of *C. auratus* while exploring a novel environment and when learning a new task. As already demonstrated in several other studies (Burgess et al. 2001; Vargas et al. 2004, 2006), goldfish can encode both non-geometric and geometric information and encode the goal location using geometrical clues (Bingman and Mench 1990; Salas et al. 1996a, b; Durán et al. 2008). In conclusion, fish needed to encode new geometric information due to the introduction of the modified third gate (Rajan et al. 2011).

An interesting question is whether animals possess self-awareness, such as recognizing themselves in a mirror (for a recent behavioural study see Kohda et al. 2019). In particular, since fish cannot self-recognize, Desjardins and Fernald (2010) asked whether fish could distinguish between fighting a mirror image and fighting a real fish. They used qRT-PCR (Quantitative Reverse Transcription Polymerase Chain Reaction) to measure mRNA expression of *egr-1* and *c-fos* in four brain regions of *A. burtoni*, i.e. the dorsomedial telencephalon, the dorsolateral telencephalon (see Fig. 4b, sections A–C), the preoptic area (see Fig. 4b, section C) and the cerebellum (see Fig. 4b, sections J, K). Fish were divided in three groups: (1) fish subjected to fighting with a conspecific male across a clear barrier (opponent group), (2) fish subjected to fighting with a mirror image (mirror group) or (3) fish without an opponent (control). No differences in aggression levels were found between ‘opponent’ and ‘mirror’ males, but ‘mirror fights’ and ‘opponent fights’ had different effects on the brain. ‘Mirror’ males had higher levels of *egr-1* expression in the dorsolateral telencephalon than ‘opponent’ males or controls, while *c-fos* expression was significantly higher in ‘opponent’ males, than in ‘mirror’ or control males. Furthermore, ‘mirror’ males had much higher *egr-1* and *c-fos* expression levels in the dorsomedial telencephalon (a potential amygdala homologue) than ‘opponent’ males or controls. This suggests that fish may experience fear when fighting their mirror image. In the cerebellum, there were no differences in *egr-1* or *c-fos* expression among any of the males. Overall, males fighting an opponent through a clear barrier or fighting their mirror image showed similar behaviour and similar gene expression in the pre-optic area and in the cerebellum but different gene expression in the dorsolateral and dorsomedial telencephalon. To explain the increase of *egr-1* activity in the dorsolateral telencephalon in ‘mirror’ males, two hypotheses were formulated. The first one assumes that in the dorsolateral telencephalon *egr-1* may operate as a transcription factor for genes involved in stress responses (for example, glucocorticoid) (Bannerman et al. 1995), indicating the encoding of “stress-related spatial information” (Desjardins and Fernald 2010). It was rejected though, as there was a simultaneous increase of *c-fos* expression in the dorsolateral telencephalon in ‘opponent’ males rather than in ‘mirror’ males. The second, more likely hypothesis assumes that the mirror image represents “a perfectly size matched opponent”, possibly inducing fear in *A. burtoni* males by not reacting in familiar ways (Desjardins and Fernald 2010).

Both inter- and intra-specific cooperative behaviours are common among animals (Dugatkin 1997; Sachs et al. 2004), i.e. two or more individuals may act together to achieve a goal that each individual cannot achieve independently (Taborsky 2007; Brosnan and de Waal 2002). Weitekamp and Hofmann (2017) examined the immunohistochemical expression of *c-fos* in the social decision-making (SDM) network, known to be involved in reward processing and in the integration of salient stimuli across vertebrates (O'Connell and Hofmann 2011, 2012; Weitekamp and Hofmann 2017), in *A. burtoni*, during cooperative territory defence behaviour (Hofmann 2003). This behaviour refers to a territorial male cooperating with another male to defend his territory from an intruder. This confers an advantage, as renegotiating boundaries usually is more expensive than cooperating with a neighbour (Getty 1987). The aim of the study was to determine how neural activation of the SDM network causes variation in cooperation with neighbours and residents and to examine whether the neural activation in specific nodes of SDM network is associated with the specific role individuals play in a cooperative context. *C-fos* expression was analysed in three subregions of the medial part of the dorsal telencephalon (potentially homologous to the mammalian basolateral amygdala), one subregion of the supracommissural nucleus of the ventral pallium (see Fig. 4b, section C) (potentially homologous to the medial amygdala/bed nucleus of the stria terminalis of mammals), in the lateral subdivision of the lateral part of the dorsal telencephalon (potentially homologous to the hippocampus) (see Fig. 4b, sections A–C), in the parvocellular (potentially homologous to the paraventricular nucleus) as well as magnocellular and gigantocellular cell groups (potentially homologous to the supraoptic nucleus (Moore and Lowry 1998; O'Connell and Hofmann 2011) of the preoptic area (see Fig. 4b, section C). Furthermore, the role of dopamine was assessed by co-labeling *c-fos* with tyrosine hydroxylase (TH), a marker of dopaminergic cells (O'Connell et al. 2011) to determine if there was an increase in activity in reward-related regions and if this increase was led by cooperative behaviour. To analyse how cooperative behaviour is correlated with neural activity in SDM networks, the researchers calculated the Engagement Index (EI), a measure of “how likely an individual is to engage in cooperative defence independent of its own size or the size of the intruder” (Weitekamp and Hofmann 2017). Results indicate that in neighbours, EI is associated with aggressive displays towards the intruder and, with the increase of EI, *c-fos* expression decreased in one subregion of the medial part of the dorsal telencephalon and in a magnocellular cell group. EI was also correlated with *c-fos* induction in dopaminergic neurons of both magnocellular and parvocellular cells groups. The magnocellular cell group is considered a potentially homologous structure to the supraotic nucleus which, in mammals, produces oxytocin (OT) involved in behaviour and social cognition (Ross and Young 2009). In the same way, the magnocellular cell group of *A. burtoni* contains isotocin (OT homolog) neurons (Huffman et al. 2012) that can mediate cooperative behaviour and can cause the increase of neural activity measured (Weitekamp and Hofmann 2017). In residents, EI was associated with an aggressive display towards the intruder; there was no up-regulation of any IEGs in any brain region assessed. Furthermore, having demonstrated that the resident modulates his aggression towards the intruder based on the behaviour of the neighbour, researchers also demonstrated that there was a negative association between the *c-fos* induction in the lateral part of the dorsal telencephalon of the resident and the neighbour’s aggression directed to the intruder. The lateral part of the dorsal telencephalon is assumed to be involved in context-dependent decision-making and social cognition (Rubin et al. 2014). Since there is a negative correlation between the aggression from the neighbour directed towards the intruder and the neural activity in the lateral part of the dorsal telencephalon of the resident, and since the resident modulates its behaviour based on its neighbour’s decision, these results suggest that the lateral part of the dorsal telencephalon plays a role in this modulation of behaviour. In conclusion, partaking of the resident male in territorial defence behaviour is based on the behaviour and size of its neighbour. Additionally, neighbour behaviour is associated with neural activity in the lateral part of the dorsal telencephalon in the resident (Weitekamp and Hofmann 2017). The neighbour also participates in territorial defence based on the perceived threat of the intruder, with a correlated activity in the preoptic area as well as in preoptic dopaminergic neurons. These results suggest that, during cooperative territory defence, neighbour and resident evaluate the presence of an intruder depending on the behavioural role they play, and this role would be associated with distinct neural activity in key nodes of the SDM network. Furthermore, the reward system may mediate the cooperation in this context (Weitekamp and Hofmann 2017).

The ability of an organism to assess numerical information and compare quantities represents an advantage for many behaviours, such as foraging, reproduction and socializing (Hager and Helfman 1991; Botham and Krause 2005; Beran et al. 2013). Although much information about numerical abilities has been collected in fish (e.g. Agrillo and Bisazza 2018), information about the neural bases underling these processes was limited to non-human primates and corvids (Nieder 2013; Ditz and Nieder 2016). Very recently though, brain regions involved in quantity discrimination processes in zebrafish were identified (Messina et al. 2020). IEGs expressions of *c-fos* and *egr-1* were analysed using RT-qPCR in different areas, i.e. the retina, the optic tectum (see Fig. 4b, sections G–L), the thalamus (see Fig. 4b, section D), the telencephalon (see Fig. 4b, sections A–C), the cerebellum (see Fig. 4b, sections J, K) and the medulla oblongata. In behavioural tests (please refer to original paper for details on testing), it was found that zebrafish preferentially chose a novel stimulus when the latter changed in numerousness but not in shape or size (Messina et al. 2020), a finding that agrees with results found for macaques (Cantlon and Brannon 2007). However, on a molecular level, IEG expression was either influenced by changes in stimulus surface area, i.e. stimulus size (retina and optic tectum) or numerousness (thalamus and telencephalon). *C-fos* expression in the zebrafish retina was positively correlated with stimulus surface area, while *egr-1* expression in the retina was not affected by an increase in surface area but increased with a decrease in surface area instead. In the optic tectum (see Fig. 4b, sections G–L), *c-fos* expression decreased with an increase, and increased with a decrease in surface area. *Egr-1* expression in the tectum increased with a decrease in stimulus surface area while an increase had no effect. In the thalamus, *c-fos* expression decreased in fish that were habituated to 3 dots and tested with 9 dots, but increased in fish habituated to 9 dots and tested with 3 dots. The results for *egr-1* expression were similar but not statistically significant. In the telencephalon, *egr-1* and *c-fos* expressions decreased with an increase in numerosity and vice versa. In the cerebellum, only a change in surface area affected *egr-1* expression, while no changes in *c-fos* expression were observed regardless of testing scenario. In the medulla oblongata, there was no clear pattern of IEG expression (Messina et al. 2020).

The impulse of organisms to socialize and approach individuals of their own species, the so-called ‘social preference behaviour’ has been found in a variety of species, including humans and zebrafish (Sloan Wilson et al. 1994). However, as in other social species, a small part of a normally raised zebrafish population will have fewer social preferences than most other individuals or may even be  aversive to social cues, i.e. exhibit a type of ‘loner’ behaviour (Sloan Wilson et al. 1994; Dreosti et al. 2015). Recently, it was tested how brain activity and behaviour are affected by social isolation (Tunbak et al. 2020) and compared between such ‘loner’ fish (anti-social fish found in the normal population) and fish that were simply deprived of social contact, termed ‘lonely’ fish. Fish (‘loners’ and ‘lonely’ fish and controls) were subdivided in additional experimental and control groups [please refer to original paper by Tunbak et al. (2020)]. Whole-brain two-photon imaging of *c-fos* expression was performed, focusing on brain structures implicated in the SBN (Social Behaviour Network), (O’Connell and Hofmann 2011). The average activity map for each rearing/sociality group was then compared to the average activity map of a sibling fish, raised under similar conditions and tested for 30 min without social cues. There were two areas where significant differences were found, i.e. the caudal hypothalamus and the preoptic area (see Fig. 4b, section C), highlighting their roles in social preference behaviour. Furthermore, *c-fos* brain maps of control and isolated fish not exposed to social clues during the experiment were compared. There was an increase in activity in the optic tectum (see Fig. 4b, sections G–L) as well as in the posterior tuberal nucleus. These structures are known to be involved in visual processing and in stress responses, respectively (McDowell et al. 2004; Ziv et al. 2013; Wee et al. 2019). Results found suggest that isolation increases visual sensitivity (activity changes in the optic tectum), as well as increased activity in the posterior tuberal nucleus (Tunbak et al. 2020). Overall, there were significant differences regarding neural activity in brain areas linked to social behaviour, social cue processing, and anxiety or stress between the groups. Short isolation increases the sensitivity to social stimuli, but the increased sensitivity to social stimuli leads to an increase of anxiety and stress levels if the isolation is prolonged. Social preference in ‘lonely’ fish could be restored by an anxiolytic drug that acts on the monoaminergic system, i.e. by reducing serotonin levels.

The expression of IEGs can be induced not only by cognitive processes, but also by a variety of other factors including pharmacological stimulation. For example, the exposure of zebrafish larvae (*Danio rerio*) to pentylenetetrazole (PTZ, a common convulsant agent) induced the expression of *c-fos* in the optic tectum (see Fig. 4b, sections D–I) and cerebellum (see Fig. 4b, sections J, K) as well as behavioural changes ending up in clonus-like convulsions (Baraban et al. 2005). Similarly, the injection of kainic acid (a glutamate receptor agonist) in *A. burtoni* altered *egr-1* expression in different regions of the diencephalon (including the anterior part of parvocellular preoptic nucleus, magnocellular preoptic nuclei, and the anterior nucleus of the thalamus) in the olfactory bulbs (see Fig. 4b, section A), the ventral nucleus of the ventral telencephalon, the central and lateral zone of the dorsal telencephalon (see Fig. 4b, sections A–C), and in the optic tectum (see Fig. 4b, sections D–I) (Burmeister et al. 2005). An interesting study combining pharmacological stimulation, IEGs expression and motivational behaviour showed that the administration of d-amphetamine increased the expression of *c-fos* in the lateral zone and the medial zone of the adult zebrafish telencephalon and that the lateral zone of the telencephalon is involved during drug-seeking behaviour (von Trotha et al. 2014). Other studies combining pharmacological stimulation and IEGs expression were conducted on rainbow trout (*Oncorhynchus mykiss*) (Matsuoka et al. 1998) and zebrafish (*Danio rerio*) (Ruhl et al. 2017).

Similar to lesion studies, Immediate Early Gene Analyses have their own shortcomings. First, one has to identify appropriate genes for the species and task in question and establish exact protocols, which can vary considerably between species. Depending on baseline gene expression levels and intra-specific variation, it may be very difficult to quantify training (learning) effects and clearly separate these effects from other, potentially confounding factors not closely related to the treatment/stimulus of interest, such as changes in motivation or stress between experimental groups and controls. A solution to this problem may be found in in vivo imaging or optogenetics studies. Another field for future research may be provided using knock-out mutants. In mice, it has already been shown that specific knock-out mutants may lack memory for socially relevant odors, while retaining spatial memory and the ability to smell in general (Wersinger et al. 2004).

In vivo imaging techniques allow behaviour to be linked to neural substrates activated in live animals. In the last few years, zebrafish and its larvae have proven to be a suitable model system to perform such in vivo imaging studies, especially for analysing whole-brain activity (Portugues et al. 2014; Preuss et al. 2014). For instance, Preuss et al. (2014) used calcium imaging to analyse how the visual system of zebrafish detects and categorizes moving objects. To select appropriate responses, such as approach or escape behaviour, knowing the size of an object is critical. Results show, that the tectum categorizes visual targets on the basis of retinally computed size information. Calcium imaging was also used by Temizer et al. (2015) to analyse how the visual system extracts information about a looming-stimulus feature, triggering escape behaviour in zebrafish. There are three areas, including the optic tectum (see Fig. 4b, sections G–L), that respond selectively to features of looming stimuli. Furthermore, through targeted laser ablations, researches also demonstrated that, to trigger the looming-escape behaviour, the optic tectum plays a critic role (Temizer et al. 2015). Using whole-brain functional imaging, it was analysed and identified how the zebrafish larvae brain collects and implements sensory information over different time scales to select appropriate behaviours (Bahl and Engert 2020; Dragomir et al. 2020). Through random dot motion stimuli, an ‘optomotor response’ was invoked, an innate behaviour to follow the direction of the perceived motion. During the decision-making process, neuronal clusters in the midbrain and hindbrain were activated. In the midbrain, including the pretectum, and in the medial parts of the reticular formation in the anterior hindbrain, there was a concentration of neurons encoding momentary sensory information, whereas in the lateral parts of the reticular formation, the dorsal raphe nucleus and the caudal interpeduncular nucleus, the dorsal part of the pretectum, the dorsal thalamus (see Fig. 4b, section D), the torus longitudinalis (see Fig. 4b, sections E, F) and the habenula (see Fig. 4b, section D) were neurons that integrated sensory evidence (Dragomir et al. 2020; Bahl and Engert 2020).

Last but not least, another technique worth mentioning is optogenetics, which refers to the ability to control and observe cellular activity through the use of light-sensitive proteins (Rost et al. 2017). It was first used to describe genetically targeted photoreceptor expression in neurons for their selective activation or inhibition with light (Deisseroth et al. 2006) and later extended to include other photosensitive proteins (Dugué et al. 2012; Miesenböck 2009). It is not difficult to understand how this technique, over the last few decades, has revolutionized the study of neuronal activity (Scanziani and Häusser 2009). It allows to perform an experiment within a specific time window and to control neuronal activity with a very high spatial–temporal resolution (Rost et al. 2017). One can manipulate neurons to verify how the manipulation alters brain circuits and changes behaviour accordingly (Rost et al. 2017). For example, the optogenetic activation of a class of interneurons in zebrafish spinal cord is sufficient to produce a coordinated swimming pattern without sensory stimuli or input from higher brain structures, thus representing the excitatory unit of the locomotor circuitry in the fish (Ljunggren et al. 2014). Optogenetic activation of the left-dorsal habenula (see Fig. 4b, section D) in eye-removed zebrafish larvae triggers innate light-preference behaviour in zebrafish larvae (Zhang et al. 2017)., This behaviour is critical for survival and highly conserved (Crozier and Pincus 1927; Gong et al. 2010; Steenbergen et al. 2011; Wang et al. 2014; Ward et al. 2008; Yamanaka et al. 2013; Zhang et al. 2017).

## Conclusion

In this review, the currently available information about the neural substrates involved in cognitive information processing in fish is summarized, giving a roadmap for future research. IEG and lesion studies have proven to be powerful and potentially complementary methods, allowing identification of brain areas underlying several cognitive aspects, specifically if used in combination. Optogenetics and in vivo studies may further complement these techniques. Scientific research in this field is still in the early stages and many interesting questions remain unanswered. While a lot is known about the fish brain in general, specific functions of many brain regions are still unknown or have only partially been exposed, specifically in regards to cognitive abilities. Lastly, both the neuroanatomy and the behaviour involved in cognitive processes have only been studied in a few representatives of the more than 33,000 extant fish species. In the future, hopefully more scientific endeavours will aim to address cognition in fish using a more doverse range of species and a more holistic approach, i.e. by not only asking whether or not an animal can perform a cognitive task, but also by trying to discover what neural substrates are involved in the processing of such a task using several if not all of the methods currently available.
